# Interaction of Fumonisin B1, *N*-Palmitoyl-Fumonisin B1, 5-*O*-Palmitoyl-Fumonisin B1, and Fumonisin B4 Mycotoxins with Human Serum Albumin and Their Toxic Impacts on Zebrafish Embryos

**DOI:** 10.3390/biom13050755

**Published:** 2023-04-27

**Authors:** Zsolt Csenki, Tibor Bartók, Illés Bock, Levente Horváth, Beáta Lemli, Balázs Zoltán Zsidó, Cserne Angeli, Csaba Hetényi, István Szabó, Béla Urbányi, Melinda Kovács, Miklós Poór

**Affiliations:** 1 Department of Environmental Toxicology, Institute of Aquaculture and Environmental Safety, Hungarian University of Agriculture and Life Sciences, Páter Károly u. 1, H-2100 Gödöllő, Hungary; 2 Fumizol Ltd., Kisfaludy u. 6/B, H-6725 Szeged, Hungary; 3 Institute of Physiology and Nutrition, Agriobiotechnology and Precision Breeding for Food Security National Laboratory, Hungarian University of Agriculture and Life Sciences, Guba Sándor út 40, H-7400 Kaposvár, Hungary; 4 Department of Pharmacology, Faculty of Pharmacy, University of Pécs, Rókus u. 2, H-7624 Pécs, Hungary; 5 Green Chemistry Research Group, János Szentágothai Research Centre, University of Pécs, Ifjúság útja 20, H-7624 Pécs, Hungary; 6 Department of Pharmacology and Pharmacotherapy, Pharmacoinformatics Unit, Medical School, University of Pécs, Szigeti út 12, H-7624 Pécs, Hungary; 7 Department of Aquaculture, Institute of Aquaculture and Environmental Safety, Hungarian University of Agriculture and Life Sciences, Páter Károly u. 1, H-2100 Gödöllő, Hungary; 8 ELKH-MATE Mycotoxins in the Food Chain Research Group, Guba Sándor út 40, H-7400 Kaposvár, Hungary; 9 Food Biotechnology Research Group, János Szentágothai Research Centre, University of Pécs, Ifjúság útja 20, H-7624 Pécs, Hungary

**Keywords:** fumonisin B1, *N*-palmitoyl-fumonisin B1, 5-*O*-palmitoyl-fumonisin B1, fumonisin B4, human serum albumin, zebrafish embryos

## Abstract

Fumonisins are frequent food contaminants. The high exposure to fumonisins can cause harmful effects in humans and animals. Fumonisin B1 (FB1) is the most typical member of this group; however, the occurrence of several other derivatives has been reported. Acylated metabolites of FB1 have also been described as possible food contaminants, and the very limited data available suggest their significantly higher toxicity compared to FB1. Furthermore, the physicochemical and toxicokinetic properties (e.g., albumin binding) of acyl-FB1 derivatives may show large differences compared to the parent mycotoxin. Therefore, we tested the interactions of FB1, *N*-palmitoyl-FB1 (*N*-pal-FB1), 5-*O*-palmitoyl-FB1 (5-*O*-pal-FB1), and fumonisin B4 (FB4) with human serum albumin as well as the toxic effects of these mycotoxins on zebrafish embryos were examined. Based on our results, the most important observations and conclusions are the following: (1) FB1 and FB4 bind to albumin with low affinity, while palmitoyl-FB1 derivatives form highly stable complexes with the protein. (2) *N*-pal-FB1 and 5-*O*-pal-FB1 likely occupy more high-affinity binding sites on albumin. (3) Among the mycotoxins tested, *N*-pal-FB1 showed the most toxic effects on zebrafish, followed by 5-*O*-pal-FB1, FB4, and FB1. (4) Our study provides the first in vivo toxicity data regarding *N*-pal-FB1, 5-*O*-pal-FB1, and FB4.

## 1. Introduction

**Figure 1:**
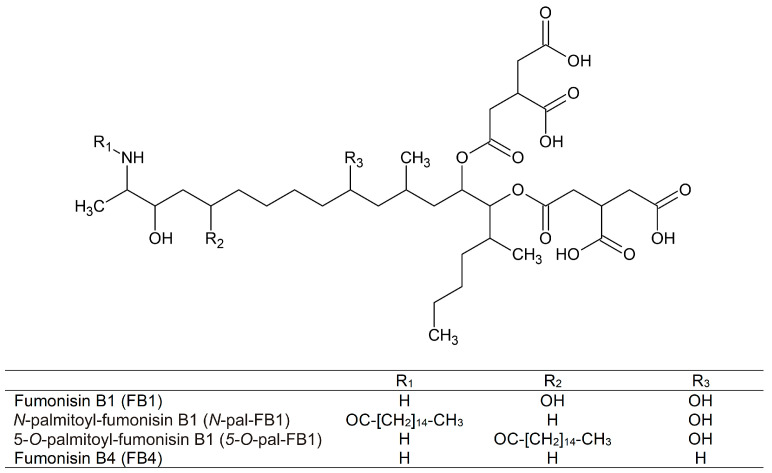
Chemical structures of fumonisin B1 (FB1), *N*-palmitoyl-fumonisin B1 (*N*-pal-FB1), 5-*O*-palmitoyl-fumonisin B1 (5-*O*-pal-FB1), and fumonisin B4 (FB4).

Fumonisins, a group of mycotoxins, are toxic metabolites of molds. As it has been reported, several *Fusarium* [1], one *Alternaria* [2], some black *Aspergillus* [3] and *Tolypocladium* [4] species are involved in the production of fumonisins. Fumonisin contamination can occur in several agricultural commodities, but their most typical occurrence is in moldy maize and maize products [5]. According to the longest running and most comprehensive survey on mycotoxin occurrence, the prevalence of fumonisins in feeds varies between 26 and 87% (on average 47%) in different regions of the world, with the highest proportion (on average 71%) in corn (DSM World Mycotoxin Survey, December 2022). Consumption of feed containing fumonisins can cause many pathophysiological problems, including equine leukoencephalomalacia [6], porcine pulmonary oedema disease [7,8], hepatocarcinogenicity [9], and nephrotoxicity [10] in rodents. Neurotoxic effects of fumonisin B1 (FB1; Figure 1) have also been reported both in vivo and in vitro [11]. In addition, recent studies demonstrated the harmful impacts of FB1 on the digestive system as well [12]. In relation to humans, the high exposure to fumonisins can be associated with the increased incidence of esophageal cancer [13,14,15] and neural tube defects [16]. A recent study performed on human esophageal epithelial cells suggests the involvement of the HDAC/PI3K/Akt pathway in FB1-induced carcinogenesis [17]. Based on the evaluation of IARC, FB1 is a possible human carcinogen (group 2B) [18]. Toxic mechanisms of FB1 show high complexity; the mycotoxin interferes with sphingolipid biosynthesis and with the immune system, and induces oxidative stress, apoptosis, and autophagy [12,19]. EU limit values (Commission Regulation 1881/2006/EC) were established (FB1 + fumonisin B2) in food and recommendation values have also been made for animal feed (Directive 2002/32/EC of the European Parliament—Council statement). Considering the chronic toxic impacts of FB1 on mice, the CONTAM Panel of EFSA established 1.0 µg/kg body weight/day as the tolerable daily intake [20]. Since the discovery of the first fumonisins (FB1 and fumonisin B2) in 1988 [21,22], thirty fumonisins have been structurally identified (groups A, B, C, and P) [1,23,24]. In addition, nearly hundred fumonisins have been detected by HPLC/MS and MS/MS techniques [25,26,27,28,29], including *O*-acyl- and *N*-acyl-FB1 derivatives [30,31,32]. The importance of *O*-acylated-FB1 toxins was further enhanced by the fact that these metabolites have also been observed in corn following natural infection in Italy [33]. *N*-acyl-FB1 derivatives have not yet been identified in agricultural raw materials; however, Harrer and coworkers reported these derivatives in rat kidneys and liver samples after the intraperitoneal treatment of the experimental animals with FB1 [34]. They also suggested the considerably higher toxicity of *N*-acyl-FB1 compared to FB1 [35]. Moreover, Park and colleagues demonstrated the presence of *N*-acyl-FB1 compounds in tortillas after nixtamalization (heating of maize in alkaline medium during tortilla productions) of maize containing FB1, while these derivatives were not contained in the original raw material [36]. Among the several published fumonisins, the toxic impacts of FB1 and fumonisin B2 have been extensively studied; however, *N*-acyl-FB1 was the sole acylated FB1 derivative tested, and its toxicity was examined only in cell cultures [35]. Currently, no data are available regarding the in vivo toxicity of *N*- and *O*-acyl-FB1 derivatives. In recent years, we have successfully isolated/produced *N*-palmitoyl-FB1 (*N*-pal-FB1), 5-*O*-palmitoyl-FB1 (5-*O*-pal-FB1), and fumonisin B4 (FB4) toxins (Figure 1) [32]. Thus, it is possible now to examine their in vivo toxicity compared to FB1.

Serum albumin forms high-affinity complexes with many xenobiotics; these molecules appear in circulation mainly in albumin-bound form, causing a depot effect [37]. The partial entrapment by albumin can result in the slower tissue uptake and/or elimination of the bound substances, affecting their pharmacokinetic/toxicokinetic properties [38]. Fatty acids (FAs) are typical, high-affinity ligands of human serum albumin (HSA). The protein can bind up to nine equivalents of long-chain FAs; these binding sites are called as FA1–FA9. [37]. There are two well-known drug binding sites on HSA, namely Sudlow’s Site I (in FA7) and Sudlow’s Site II (in FA3–FA4). Moreover, the presence of a further drug binding site, the Heme pocket (in FA1) had also been emphasized [37,39].

Zebrafish embryo is a widely applied vertebrate model for studying developmental toxicity of different xenobiotics [40,41,42], such as mycotoxins [43]. The visual investigation of the externally fertilized and transparent embryos is relatively simple; therefore, they are useful in the evaluation of xenobiotic-induced toxicity/mortality and developmental abnormalities in the different larval stages [44,45]. Moreover, their embryogenesis shows high similarity to higher vertebrates [46]. The FB1-induced toxic impacts have been previously assessed in mixtures with zearalenone [47] or aflatoxin B1 [48] on zebrafish embryos, where enzyme induction and increased oxidative stress were described. Furthermore, the mixture of aflatoxin B1 and FB1 caused significant reduction in growth performance in juvenile North African catfish (*Clarias gariepinus*) [49].

Considering the high stability of palmitic acid–albumin complexes [50,51], it is reasonable to assume the relevant interaction of palmitoyl derivatives of FB1 with the protein. Thus, the interactions of FB1, *N*-pal-FB1, 5-*O*-pal-FB1, and FB4 were examined with HSA using fluorescence spectroscopic, ultrafiltration, ultracentrifugation, and modeling studies. Furthermore, we hypothesized that *N*-pal-FB1 and 5-*O*-pal-FB1 may exert higher acute toxicity than FB1 and FB4. To confirm or reject this idea, the toxic impacts of FB1, *N*-pal-FB1, 5-*O*-pal-FB1, and FB4 were tested on zebrafish embryos to evaluate mycotoxin-induced mortality and sublethal toxic effects.

## 2. Materials and Methods

### 2.1. Reagents

Fumonisin B1 (FB1), *N*-palmitoyl-fumonisin B1 (*N*-pal-FB1), 5-*O*-palmitoyl-fumonisin B1 (5-*O*-pal-FB1), and fumonisin B4 (FB4) were provided by Fumizol Ltd. (Szeged, Hungary). N-C17:0-FB1 and FB1-^13^C34 internal standards were from Fumizol Ltd. and Romer Labs (Tulln, Austria), respectively. Human serum albumin (HSA), palmitic acid, warfarin, naproxen, *S*-camptothecin, and tricaine methanesulfonate (MS-222) were obtained from Merck (Darmstadt, Germany). FB1, *N*-pal-FB1, 5-*O*-pal-FB1, palmitic acid, and FB4 (each 5 mM) were dissolved in dimethyl sulfoxide; these solutions were applied in spectroscopic, ultracentrifugation, and ultrafiltration studies.

### 2.2. Spectroscopic Experiments

Spectra were collected at 25 °C, employing an F-4500 fluorometer (Hitachi, Tokyo, Japan) and a V730 UV-Vis photometer (Jasco, Tokyo, Japan). The effects of mycotoxins and palmitic acid (final concentrations: 0.0, 0.5, 1.0, 2.0, 3.0, 5.0, and 10.0 μM) on the emission spectrum of HSA (2 μM) were tested in phosphate-buffered saline (PBS, pH 7.4), where changes in the emission signals of the protein were evaluated at 340 nm. Based on the UV-Vis spectra, inner-filter effects of ligand molecules were corrected [52,53].

### 2.3. Ultracentrifugation Studies

Albumin and HSA-bound fraction of mycotoxins were sedimented with ultracentrifugation using the previously reported method [54,55]. Thereafter, the free fraction of ligand molecules was quantified (see details in the following paragraph). In the first experiment, samples contained FB1, *N*-pal-FB1, 5-*O*-pal-FB1, or FB4 (each 10 μM) in the presence of HSA (40 g/L). In regard to *N*-pal-FB1 and 5-*O*-pal-FB1, experiments were also performed in the presence of 2, 5, and 10 μM of HSA.

FB1, *N*-pal-FB1, 5-*O*-pal-FB1, and FB4 were quantified by LC–MS applying the following conditions: Samples were analyzed using a 1100 HPLC (Agilent, Santa Clara, CA, USA) with a 1946D ESI-MS (Agilent). The HPLC system consisted of a vacuum degasser, a binary pump, a well-plate sampler, and a column thermostat set to 40 °C. Gradient separation was performed on a Security Guard column (C18; Phenomenex, Torrance, CA, USA) and a Kinetex C18 (50 × 4.6 mm, 2.6 µm; Phenomenex) analytical column (flow rate: 0.8 mL/min). Both solvent A (water) and solvent B (acetonitrile, ACN) contained 0.1 *v/v*% of formic acid. The gradient started with 24% B and increased linearly to 80% in 14 min, which increased to 100% in 1 min, then held for 2 min, after which it returned to the initial value in 2 min followed by holding this value for 4 min. The analysis was started with an injector programme, including the addition of 1 µL internal standard mixture (1 ng FB1-^13^C34 and 1 ng N-C17:0-FB1 in ACN-water (1:1 *v*/*v*%) to each 1 µL sample. The mass spectrometer (MS) was set to the following parameters: nebulizer gas (N_2_) pressure, 50 psi; drying gas (N_2_) flow rate, 12 L/min and temperature, 350 °C; capillary high voltage, 3000 V. MS data was acquired in positive-ion SIM (selective ion monitoring) mode: 0–8 min: 690, 722, 756 *m/z*; 8–15 min: 961, 975 *m/z*. Stock solutions for calibration (FB1, *N*-pal-FB1, 5-*O*-pal-FB1, and FB4) were freshly prepared from vials containing 100 µg of these substances by dissolution with ACN-water (1:1 *v*/*v*%). These solutions were further diluted with the same solvent. The following standards were prepared from FB1 and FB4: 100, 50, 10, 5, 1, 0.5, 0.2, 0.1, and 0.05 ng/µL. The following standards were prepared from *N*-pal-FB1 and 5-*O*-pal-FB1: 50, 25, 5, 2.5, 0.5, 0.25, 0.1, 0.05, and 0.025 ng/µL. The linearity (R^2^), limit of detection (LOD; 3:1 signal/noise ratio), and limit of quantification (LOQ; 10:1 signal/noise ratio) of the components were as follows: FB1 (lin: 0.1–100 ng/µL, R^2^ = 0.9998; LOD = 50 pg/µL; LOQ = 100 pg/µL); FB4 (lin: 0.1–100 ng/µL, R^2^ = 0.9999; LOD = 50 pg/µL; LOQ = 100 pg/µL); *N*-pal-FB1 (lin: 0.025–50 ng/µL, R^2^ = 0.9989; LOD = 10 pg/µL; LOQ = 25 pg/µL); and 5-*O*-pal-FB1 (lin: 0.025–50 ng/µL, R^2^ = 0.9999; LOD = 10 pg/µL; LOQ = 25 pg/µL).

Binding constants of FB1–HSA and FB4–HSA were calculated as it has been previously reported [55], assuming 1:1 stoichiometry of mycotoxin-albumin complexes.

### 2.4. Ultrafiltration Studies

The impacts of FB1, *N*-pal-FB1, 5-*O*-pal-FB1, palmitic acid, and FB4 were tested on the albumin binding of the Site I ligand warfarin, the Site II ligand naproxen, and the FA1 site ligand *S*-camptothecin, employing the previously reported ultrafiltration methods [55,56]. Concentrations of warfarin, naproxen, and *S*-camptothecin in the supernatants were determined with HPLC, as it has been earlier described [56]. Statistical differences were assessed based on one-way ANOVA and Tukey post-hoc tests, employing SPSS Statistics software (version 21; IBM, Armonk, NY, USA) (* *p* < 0.05, ** *p* < 0.01).

### 2.5. Modeling Studies

The structures of *N*-pal-FB1 and 5-*O*-pal-FB1 were built in Maestro (Schrödinger Maestro Schrödinger Release 2020-4). According to the IUPAC nomenclature, the chirality centers of the ligands were built as follows: (2S,2′S)-2,2′-[(5S,6R,7R,9R,11S,16R,18S,19S)-19-amino-11,16,18-trihydroxy-5,9-dimethylicosane-6,7-diyl]bis[oxy(2-oxoethane-2,1-diyl)]dibutanedioic acid. The energy minimization of the ligands was performed with OpenBabel [57] with subsequent steepest descent and conjugate gradient minimizations. Then, the resultant structures were further subject to MOPAC [58] energy minimization with a PM7 parametrization [59]. The gradient norm was set to 0.001. In AutoDock Tools [60], the Gasteiger–Marsilli [61] partial charges were assigned to the ligand atoms. Flexibility of the ligands was allowed at all active torsions.

Similar to our earlier studies, atomic coordinates of human albumin (PDB code: 1ao6) were from the Protein Data Bank [62,63,64]. The target molecule was equipped with polar hydrogen atoms and Gasteiger–Marsilli partial charges in AutoDock Tools.

Docking of ligands to HSA was performed applying AutoDock 4.2.6 [60] (number of grid points: 100 × 100 × 100; grid spacing: 0.803 Å). Blind docking was carried out, where the whole surface of the target molecule was covered by the docking box. Global search was executed using the Lamarckian genetic algorithm. After hundred docking runs, ligand conformations were ranked based on the interaction energy values calculated [65]. Subsequent evaluations and the collection of the interacting amino acids were also carried out [66].

### 2.6. Testing the Toxic Impacts of Fumonisins on Zebrafish Embryos

For testing of toxic effects on zebrafish embryos, we used laboratory-reared AB zebrafish line. The fish were kept in a recirculating aquaculture system (Tecniplast S.p.a., Italy, Buguggiate) at controlled microenvironmental conditions (temperature: 25.5 ± 0.5 °C, conductivity: 550 ± 50 μS/cm, pH: 7.0 ± 0.2) with a 14 h:10 h light/dark cycle.

On the previous day of spawning, fish were placed in mating tanks with the males and females separated by a divider. On the next day, the dividers were removed, and spawning was initiated once the lights turned on. The eggs were collected and rinsed with system water and were kept in sterile E3 medium (5 mM NaCl, 0.33 mM CaCl_2_, 0.17 mM KCl, 0.33 mM MgSO_4_) [67] and incubated at 25.5 ± 1.0 °C until the start of the experiments. Once a day, coagulated embryos were discarded, and the medium was refreshed.

To test the toxic effects of fumonisins, we performed a Zebrafish Embryo Toxicity Assay (ZETA) [68], which is a slightly modified version of the standard OECD Fish Embryo Acute Toxicity (FET) test [69]. At 96 hpf (hours past fertilization), the zebrafish embryos were exposed for 24 h to the following concentrations of each compound: 0.78, 1.56, 3.12, 6.25, 12.5, 25, 50, 100, and 200 μM. Stock solution of each compound (1000 mg/mL) was made using dimethyl sulfoxide solvent, then the treatment solutions were prepared by diluting it in E3 medium. Embryos were transferred to 24-well tissue-culture plates (JET Biofil; Guangzhou, China) in groups of five in four replicates. Each well was filled with 2 mL of treatment solutions as well as E3 medium control and solvent control at a respective concentration. The plates were placed in an incubator at 25.5 ± 1.0 °C. After 24 h, mortalities and morphological deformities were checked under a dissecting microscope (Leica Microsystems GmbH; Wetzlar, Germany).

Surviving embryos were anesthetized using 0.02% MS-222 solution, then were transferred into Petri dishes containing 5% methylcellulose solution. Lateral bright field images (exposure time: 6 ms, magnification: 30×) were captured using a Leica M205 FA stereomicroscope equipped with a Leica DFC 7000T camera and Leica Application Suite X software (Leica Microsystems GmbH; Wetzlar, Germany).

For the statistical analysis of lethal and nonlethal effects, we applied two-way ANOVA with Tukey’s multiple comparisons test (*p* < 0.0001). The analysis was carried out using GraphPad Prism 9 software (GraphPad Software, Boston, MA, USA).

## 3. Results

### 3.1. Effects of Fumonisins and Palmitic Acid on the Emission Spectrum of HSA

In these experiments, emission spectra of HSA (2 μM) were recorded (λ_ex_ = 295 nm) without and with mycotoxins (0–10 μM) in PBS (pH 7.4). The effects of *N*-palmitoyl and 5-*O*-palmitoyl FB1 derivatives were also compared with the impacts of palmitic acid.

**Figure 2:**
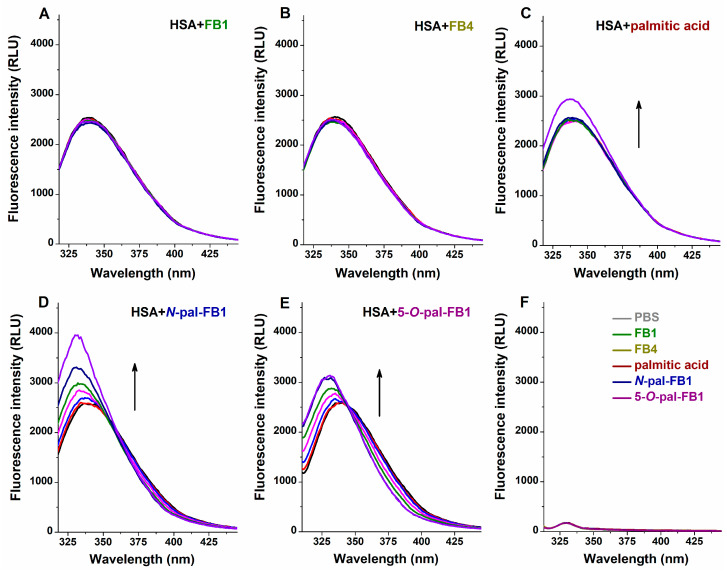
Effects of FB1 (**A**), FB4 (**B**), palmitic acid (**C**), *N*-pal-FB1 (**D**), and 5-*O*-pal-FB1 (**E**) (0.0, 0.5, 1.0, 2.0, 3.0, 5.0 and 10.0 μM) on the emission spectrum of HSA (2.0 μM) in PBS (pH 7.4; λ_ex_ = 295 nm). FB1, FB4, *N*-pal-FB1, 5-*O*-pal-FB1, and palmitic acid (each 10 μM) did not show fluorescence in the absence of HSA (**F**).

FB1 (Figure 2A) and FB4 (Figure 2B) did not induce considerable changes in the emission spectrum of albumin. Only the highest concentration (10 μM) of palmitic acid caused an increase in the fluorescence of HSA (Figure 2C). However, both *N*-pal-FB1 (Figure 2D) and 5-*O*-pal-FB1 (Figure 2E) gradually increased the fluorescence intensity of HSA, accompanied with blueshifts (*N*-pal-FB1: 340 nm → 330 nm; 5-*O*-pal-FB1: 340 nm → 329 nm) in the emission wavelength maximum of the protein. Importantly, mycotoxins and palmitic acid did not show fluorescence in the absence of albumin (Figure 2F), and they have no absorption at the wavelength range used.

### 3.2. Interaction of Fumonisins with HSA Based on Ultracentrifugation Studies

**Figure 3:**
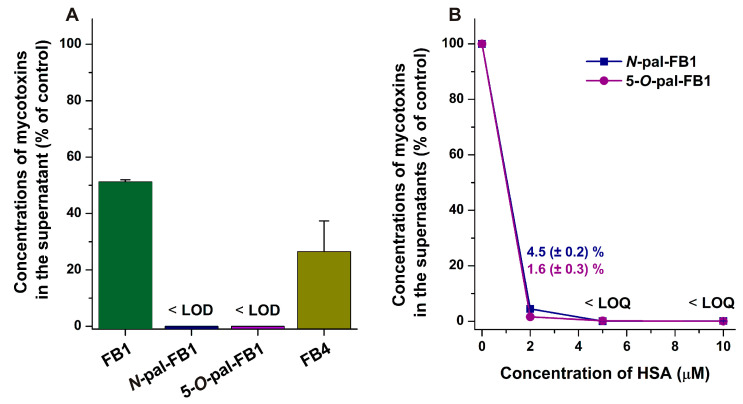
Concentrations of mycotoxins (±SEM) in the supernatants after ultracentrifugation (16 h, 170,000 *g*, 20 °C; PBS, pH 7.4). FB1, *N*-pal-FB1, 5-*O*-pal-FB1, or FB4 (each 10 μM) were ultracentrifuged with 40 g/L of HSA (**A**). Thereafter, palmitoyl derivatives were also centrifuged with 2, 5, or 10 μM HSA (**B**). After albumin was ultracentrifuged without the addition of mycotoxins, no fumonisins were detected in the supernatants. Absolute concentrations of mycotoxins in the control samples (fumonisins were centrifuged without albumin) were 9.8 ± 0.1 μM for FB1, 10.0 ± 0.3 μM for FB4, 8.7 ± 0.9 μM for *N*-pal-FB1, and 8.2 ± 0.2 μM for 5-*O*-pal-FB1.

Ultracentrifugation experiments were also carried out, where samples contained FB1, FB4, *N*-pal-FB1, or 5-*O*-pal-FB1 (each 10 μM) with or without HSA (40 g/L ≈ 600 μM approximates the concentration of albumin in circulation). As Figure 3 demonstrates, albumin decreased the amounts of *N*-pal-FB1 and 5-*O*-pal-FB1 below LOD, while approximately 50% and 75% decreases in FB1 and FB4 levels were observed, respectively (Figure 3A). Based on these data, the binding constants of FB1–HSA (*K* = 1.6 × 10^3^ L/mol) and FB4–HSA (*K* = 6.6 × 10^3^ L/mol) were determined assuming 1:1 stoichiometry of mycotoxin–albumin complexes [55].

Considering the strong removal of palmitoyl-FB1 derivatives by HSA, we repeated these experiments with 2, 5, and 10 μM of HSA. Interestingly, even 2 μM of the protein led to remarkable decreases in the concentrations of *N*-pal-FB1 and 5-*O*-pal-FB1 in the supernatant (Figure 3B).

### 3.3. Impacts of Fumonisins and Palmitic Acid on the Interactions of Site Markers with HSA

**Figure 4:**
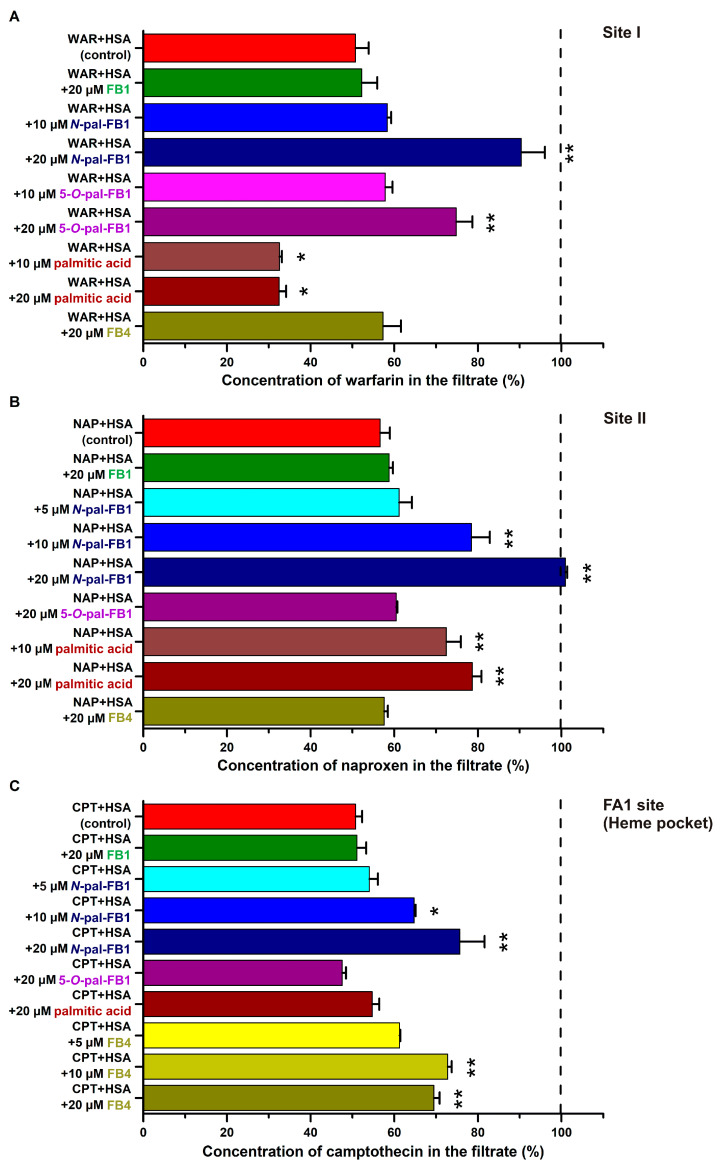
Effects of FB1, *N*-pal-FB1, 5-*O*-pal-FB1, palmitic acid, and FB4 on the concentrations (% ±SEM) of warfarin (WAR; (**A**)), naproxen (NAP; (**B**)), and *S*-camptothecin (CPT; (**C**)) in the filtrate (PBS; * *p* < 0.05, ** *p* < 0.01; see further experimental details in Section 2.4). In each model, 100% (see with black dashed line) was the level of the site marker in the filtrate when the site marker was filtered without the protein.

To examine the binding sites and to test the impacts of fumonisins on site marker–HSA interactions, ultrafiltration experiments were performed. The filtered fractions of the site markers tested were not influenced by FB1 (Figure 4). Furthermore, the albumin binding of warfarin and naproxen were not affected by FB4; however, even in its lower concentrations (5 and 10 μM), FB4 moderately increased the *S*-camptothecin levels in the filtrate (Figure 4C).

Palmitic acid decreased the filtered fraction of warfarin (Figure 4A). Interestingly, *N*-pal-FB1 and 5-*O*-pal-FB1 showed opposite impacts: Their higher concentration (20 μM) markedly elevated warfarin levels in the filtrates (Figure 4A).

Palmitic acid and *N*-pal-FB1 caused moderate and large elevation in the filtered fraction of naproxen, respectively (Figure 4B). However, 5-*O*-pal-FB1 did not affect the naproxen–HSA interaction.

In addition, palmitic acid and 5-*O*-pal-FB1 did not change the albumin binding of *S*-camptothecin, while *N*-pal-FB1 induced the concentration-dependent elevation in the filtered fraction of the FA1 site marker (Figure 4C).

### 3.4. Molecular Modeling Studies

**Figure 5:**
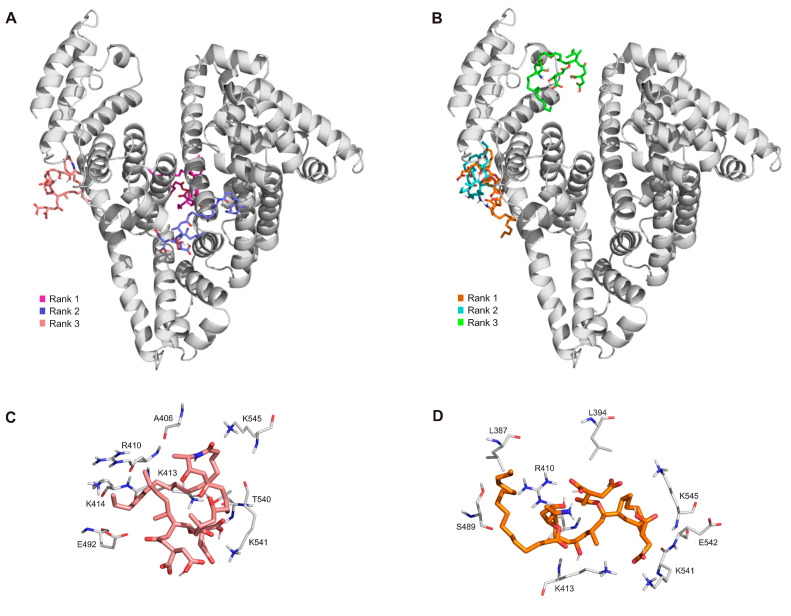
The first three ranked binding modes of *N*-pal-FB1 (**A**) and 5-*O*-pal-FB1 (**B**) on albumin (grey cartoon). The third ranked binding mode of *N*-pal-FB1 ((**C**), pink sticks) and the first ranked binding mode of 5-*O*-pal-FB1 ((**D**), orange sticks), where the interacting amino acids were marked with thick grey lines.

The binding modes of the two palmitoyl-FB1 derivatives to HSA were examined with blind docking calculations. Based on the 100 blind docking runs for each, our results suggest that their binding sites are at the entrance of Site II (Figure 5): in the first rank for 5-*O*-pal-FB1 and in the third rank for *N*-pal-FB1. Furthermore, *N*-pal-FB1 also has two top-ranked binding modes near to Site I.

At the entrance of Site II, *N*-pal-FB1 forms interactions with R410, K413, K414, E492, K541, and K545 amino acids (Figure 5C). In blind docking studies, the first ranked binding mode of 5-*O*-pal-FB1 was found 6.2 Å from the experimental binding mode of diazepam (a known ligand of Site II), where the complex is stabilized by hydrophilic (R410 and E542), hydrophobic (L387 and L394), and ionic (K541 and K545) interactions (Figure 5B,D).

### 3.5. Toxic Impacts of Fumonisins on Zebrafish Embryos

**Figure 6:**
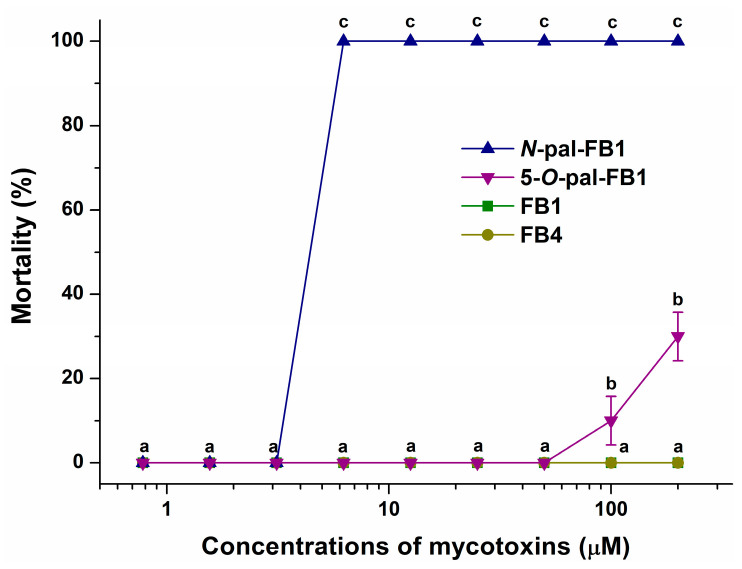
Mortality rates (% ± SEM) of zebrafish embryos at 120 hpf treated in an exposure window of 96–120 hpf with FB1, *N*-pal-FB1, 5-*O*-pal-FB1, and FB4. Statistical significance between treatment groups was established using two-way ANOVA with Tukey multiple comparisons tests (a, b, c: *p* < 0.0001).

After 24 h treatment (between 96 hpf and 120 hpf) of zebrafish embryos, no mortality was observed in E3 medium and solvent controls. Furthermore, FB1 and FB4 did not cause mortality even at 200 µM concentrations (Figure 6); however, the treatment with 100 µM and 200 µM of 5-*O*-pal-FB1 led to 10% and 30% mortality, respectively. In addition, *N*-pal-FB1 induced 100% mortality even at 6.25 µM concentration (Figure 6).

**Figure 7:**
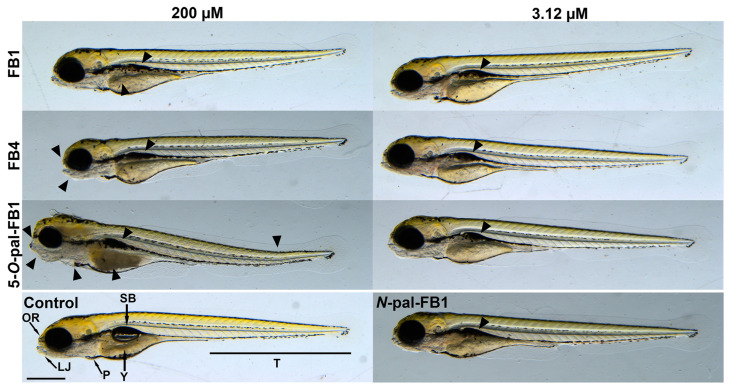
Representation of the typical malformations (black arrowheads) in 120 hpf zebrafish embryos after 24 h exposure to FB1, FB4, 5-*O*-pal-FB1, and *N*-pal-FB1 (OR, olfactory region; LJ, lower jaw; P, pericardium; T, tail; SB, swim bladder; Y, yolk; scale bar: 500 μm).

Sublethal effects of FB1, FB4, and 5-*O*-pal-FB1 are demonstrated at their 3.12 µM and 200 µM concentrations (Table 1). Since sublethal impacts can be tested only on the living zebrafish embryos, and *N*-pal-FB1 caused 100% mortality even at 6.25 µM concentration, the *N*-pal-FB1-induced malformations were evaluated only at 3.12 µM (Table 1). Uninflated swim bladder (Figure 7) was the only sublethal effect induced by 3.12 µM concentration of fumonisins tested; it was most frequently caused by *N*-pal-FB1 (80%), followed by 5-*O*-pal-FB1 (50%), FB4 (30%), and FB1 (25%).

At 200 µM concentration, FB1, FB4, and 5-*O*-pal-FB1 caused uninflated swim bladder in most of the embryos (Table 1), where the highest abundance of this phenotype was induced by 5-*O*-pal-FB1 (100%). Abnormal yolk coloration (Figure 7) was observed after the treatment with 200 µM of FB1 (15%; not statistically significant), FB4 (40%), and 5-*O*-pal-FB1 (100%). FB1 did not cause head malformations (deformations of the lower jaw and the olfactory region; Figure 7), while these deformities occurred in zebrafish embryos exposed to 200 µM of FB4 (40%) and 5-*O*-pal-FB1 (100%). Curvature of the body axis (71%) and edemas (15%; not statistically significant) were induced only by 5-*O*-pal-FB1 treatment (200 µM; Figure 7 and Table 1).

## 4. Discussion

FB1 and other fumonisins are frequent contaminants in foodstuffs and feed [70], in which the presence of *N*- and *O*-acyl derivatives have also been reported. Since fatty acids (including palmitic acid) bind to serum albumin with very high affinity, we assumed that palmitoyl-FB1 derivatives also form stable complexes with this protein. Furthermore, FB1 and FB4 are relatively hydrophilic mycotoxins while *N*-pal-FB1 and 5-*O*-pal-FB1 are highly lipophilic. Therefore, toxicokinetic and toxicodynamic properties of palmitoyl-FB1 derivatives and FB1 are likely different.

HSA includes only one single tryptophane amino acid (Trp-214), which is the most important residue regarding the fluorescence exerted by the protein [71]. Since microenvironmental changes can strongly affect the emission signal of Trp-214, ligand–albumin interactions usually decrease the fluorescence intensity of the protein, which gives the basis of fluorescence quenching studies [72]. The negligible impacts of FB1 and FB4 on the emission signal of albumin suggest that they do not form or form only barely stable complexes with HSA. Interestingly, *N*-pal-FB1 and 5-*O*-pal-FB1 increased the fluorescence emission of HSA; however, using these data, we were not able to calculate the binding constants of fumonisin–albumin complexes with the Hyperquad2006 software [53]. Nevertheless, it is reasonable to hypothesize that the spectral changes induced by *N*-pal-FB1 and 5-*O*-pal-FB1 resulted from their interactions with the protein.

With ultracentrifugation, HSA and the albumin-bound molecules can be sedimented [54,55], after which the unbound fraction of mycotoxins can be quantitatively analyzed in the supernatants. In agreement with fluorescence quenching studies, these data confirm the formation of low-affinity FB1–HSA (*K* = 2 × 10^3^ L/mol) and FB4–HSA (*K* = 7 × 10^3^ L/mol) complexes. However, even very low amounts of HSA strongly decreased the concentrations of *N*-pal-FB1 and 5-*O*-pal-FB1 in the supernatants. Albumin has certain pseudo-enzymatic activities [37]; therefore, one possible explanation is the HSA-catalyzed cleavage of palmitoyl groups. Nevertheless, we did not detect any FB1 in these samples. Therefore, it is reasonable to hypothesize the highly stable interactions of *N*-pal-FB1 and 5-*O*-pal-FB1 with the protein. In addition, even 2 μM HSA caused a very large decrease in the free unbound concentrations of palmitoyl derivatives, suggesting that *N*-pal-FB1 and 5-*O*-pal-FB1 occupy more high-affinity binding sites on albumin. Therefore, we could not calculate the binding constants of *N*-pal-FB1–albumin and 5-*O*-pal-FB1–albumin complexes from these data. In agreement with these results, high-resolution X-ray crystallographic and two-dimensional nuclear magnetic resonance analyses demonstrated many binding sites of palmitic acid on HSA, including three high-affinity binding sites in FA2, FA4, and FA5 [73,74].

We also tested the impacts of fumonisins and palmitic acid on the albumin binding of site marker drugs applying ultrafiltration technique. Since HSA (with the site markers bound) is retained by the filter units with 30 kDa (or lower) MWCO values, the elevated level of a site marker in the filtrate demonstrates its displacement from HSA or its reduced binding affinity, while a decrease in the filtered fraction of a site marker means its increased binding affinity toward the protein [53,56]. In agreement with spectroscopic and ultracentrifugation studies, the filtered fractions of warfarin and naproxen were not influenced by FB1 and FB4, while FB4 only moderately changed the filtered concentration of *S*-camptothecin (Figure 4). Palmitic acid decreased the concentration of warfarin in the filtrate (Figure 4A), which is in accordance with earlier studies where this fatty acid also increased the binding affinity of warfarin toward HSA [53,75]. Furthermore, palmitic acid moderately elevated the concentration of naproxen in the filtrate (Figure 4B). *N*-pal-FB1 caused strong (warfarin and naproxen) and moderate (*S*-camptothecin) increases in the free fractions of site markers, representing that *N*-pal-FB1 can interfere with each binding site examined (Figure 4). On the other hand, 5-*O*-pal-FB1 affected only the albumin binding of warfarin (Figure 4A), thus 5-*O*-pal-FB1 can also interfere with the warfarin–HSA interaction. These observations demonstrated the complex modulation of drug–albumin interactions by *N*-pal-FB1 and 5-*O*-pal-FB1. Interestingly, palmitoyl-FB1 derivatives produced different effects compared to FB1 or palmitic acid, suggesting their distinct binding sites and/or binding positions on HSA.

Based on blind docking studies, the third rank binding site for *N*-pal-FB1 was at the entrance of Site II (Figure 5A,C), which enables the displacement of naproxen, as it was observed in ultrafiltration experiments (Figure 4B). Furthermore, the other two top ranked binding modes of *N*-pal-FB1 were near to Site I, explaining the displacement of warfarin (Figure 4A). Molecular modeling suggested the top ranked binding sites of 5-*O*-pal-FB1 in Site II; however, in ultrafiltration experiments, the filtered fraction of naproxen was not influenced by 5-*O*-pal-FB1 (Figure 4B).

To avoid early teratogenic effects, zebrafish embryos were treated with fumonisins in a 24 h time window between 96 hpf and 120 hpf [76]. Until 96 hpf, all major organs are developed, thus the response to toxicants reflects adult physiology [77]. Furthermore, at 96 hpf, the mouth of the embryos opens which provides another potential pathway for toxicants to enter the organism. No mortality was observed after exposure to FB1 and FB4. High levels (100 and 200 μM) of 5-*O*-pal-FB1 led to 10–30% mortality, while *N*-pal-FB1 resulted in 100% mortality at 6.25 μM concentration (Figure 6). These data underline the much higher toxicological potency of palmitoyl-FB1 derivatives vs. FB1, and also demonstrate that *N*-pal-FB1 is highly toxic even compared to 5-*O*-pal-FB1. In agreement with the mortality data, sublethal effects also suggest the following order in toxicity: *N*-pal-FB1 > 5-*O*-pal-FB1 > FB4 > FB1 (Table 1). FB1 can cause a wide range of teratogenic effects such as fetal death, malformations, and growth retardation [78]. In previous studies, hydrocephalus, ossification, and short/wavy ribs occurred in mouse embryos after feeding pregnant mice for 11 days with 100 mg/kg FB1 [79], while rat embryos showed no significant teratogenic effects after feeding pregnant rats for 17 days with 15 mg/kg FB1 [80]. In the study of Azman et al. [81], 4 hpf zebrafish embryos were exposed to FB1 at 28 ± 2 °C (highest concentration: 1.4 µM), after which spinal curvature, tail malformation, and pericardial edema were observed in 96 hpf and 120 hpf embryos. We found the same sublethal effects in zebrafish embryos after the treatment with 200 µM of 5-*O*-pal-FB1. Nevertheless, in our experiment, embryos were exposed from 96 hpf to 120 hpf, which excludes early developmental effects that might be more severe. In another study performed on zebrafish embryos (exposed from 4 hpf to 96 hpf), the 96 h LC_50_ value of FB1 was 384 µM [82], which correlates with our results indicating the low acute toxicity of the parent mycotoxin.

## 5. Conclusions

In summary, we examined the interactions of mycotoxins FB1, *N*-pal-FB1, 5-*O*-pal-FB1, and FB4 with HSA applying fluorescence spectroscopic, ultracentrifugation, ultrafiltration, and modeling studies. In addition, the toxic impacts of these mycotoxins were tested on zebrafish embryos, including the mycotoxin-induced mortality and sublethal effects. Based on our results, the binding constants of FB1–HSA and FB4–HSA complexes are low, while palmitoyl-FB1 derivatives bind to the protein with high affinity. Furthermore, *N*-pal-FB1 and 5-*O*-pal-FB1 likely occupy more high-affinity binding sites on albumin. Interestingly, palmitoyl-FB1 derivatives showed different impacts on the bound fractions of site markers than palmitic acid. *N*-pal-FB1 caused 100% mortality at 6.25 μM concentration, while even the very high levels (100 μM and 200 μM) of the other mycotoxins tested induced lower (5-*O*-pal-FB1) or zero (FB1 and FB4) mortality. Considering the sublethal effects on zebrafish embryos, the order in the toxicity of these mycotoxins is the following: *N*-pal-FB1 > 5-*O*-pal-FB1 > FB4 > FB1. Our results underline that *N*-pal-FB1 and 5-*O*-pal-FB1 bind to serum albumin with much higher affinity than FB1; therefore, the toxicokinetic properties of palmitoyl derivatives may show large differences compared to the parent mycotoxin. Finally, the current report provides the first in vivo toxicity data regarding *N*-pal-FB1, 5-*O*-pal-FB1, and FB4. Considering the above-listed observations, acyl derivatives of fumonisins may have high toxicological importance.

## Figures and Tables

**Table 1:** Developmental defects (% ±SEM) in zebrafish embryos after 24 h exposure with FB1, FB4, 5-*O*-pal-FB1 and *N*-pal-FB1.

		FB1		FB4		5-*O*-Pal-FB1		*N*-pal-FB1	Control
		200 µM	3.12 µM	200 µM	3.12 µM	200 µM	3.12 µM	3.12 µM	
**Freq.** **(%)**	**USB**	85.0 ± 5.0 *	25.0 ± 9.6 *	75.0 ± 5.0 *	30.0 ± 10.0 *	100.0 ± 0.0 *	50.0 ± 5.8 *	80.00 ± 8.2 *	0.0 ± 0.0
	**AYC**	15.0 ± 5.0	–	40.0 ± 8.2 *	–	100.0 ± 0.0 *	–	–	0.0 ± 0.0
	**HM**	–	–	30.0 ± 5.8 *	–	81.3 ± 12.0 *	–	–	0.0 ± 0.0
	**CB**	–	–	–	–	70.8 ± 2.4 *	–	–	0.0 ± 0.0
	**ED**	–	–	–	–	14.6 ± 8.6	–	–	0.00 ± 0.0

Significant differences compared to the control were tested using two-way ANOVA with Tukey multiple comparisons tests (* *p* < 0.0001). USB, uninflated swim bladder; AYC, abnormal yolk coloration; HM, head malformations; CB, curvature of the body axis; ED, edema.

## Data Availability

Data will be made available upon request.
